# Value of CA-125 Glycoprotein in Predicting Acute Appendicitis; a Diagnostic Accuracy Study

**Published:** 2019-04-06

**Authors:** Mahboub Pouraghaei, Kavous Shahsavarinia, Farzad Kakaei, Sevda Gholipour-Khalili, Babak Mohammadpour, Payman Moharamzadeh, Moloud Balafar

**Affiliations:** 1 Emergency Medicine Research Center, Tabriz University of Medical Sciences, Tabriz, Iran.; 2 Department of Surgery and Transplantation, Tabriz University of Medical Sciences, Tabriz, Iran.; 3 Faculty of Medicine, Tabriz University of Medical Sciences, Tabriz, Iran.

**Keywords:** Appendix, appendicitis, CA-125 antigen, biomarkers, abdominal pain

## Abstract

**Introduction:**

Carcinogen antigen 125 (CA-125) is a glycoprotein antigen, which has shown potentials in predicting peritoneal inflammation. The aim of this study is to determine the value of CA-125 in predicting acute appendicitis (AA).

**Methods:**

This prospective diagnostic accuracy study was conducted on 15 – 70 year-old patients with acute abdominal pain, suspected to AA, referred to emergency department. The serum level of CA-125 was measured for all patients before appendectomy and its screening characteristics in detection of AA case (confirmed by histology findings) were calculated and reported with 95% confidence interval (CI).

**Results:**

95 patients with the mean age of 31.65 ± 12.9 (15-75) years were studied (54.3% male). Based on the histologic findings, 72 (75.8%) cases were categorized as AA (23 cases as severe). AA and non-AA (NAA) groups were similar regarding the mean age (p = 0.59), mean duration of symptoms (p = 0.08), mean white blood cell (WBC) count (p = 0.37), and mean PMN percentage (p = 0.55). Mean CA-125 level was 16.5 ± 20.0 U/mL in the AA group and 30.5 ± 6.1 U/mL in the NAA group (p = 0.001). Adjustment of analysis based on gender revealed a significant correlation between CA-125 level and diagnosis of AA only in females (34.23 ± 39 U/mL in NAA versus 20.7 ± 26.7 U/mL in AA, p = 0.012). The area under the ROC curve of CA-125 was 0.62 (95%CI: 0.51 to 0.72). Sensitivity, specificity, NPV, PPV, NLR, and PLR of CA-125 in 16.4 U/mL cut off (best point) were 77.8% (95%CI: 66.4 - 86.7), 50.0% (95%CI: 28.2 - 71.8), 83.6% (95%CI: 76.7 - 88.7), and 40.7% (95%CI: 27.4 - 55.6), 0.44 (95%CI 0.2 - 0.8), and 1.56 (95%CI: 1.0 - 2.4), respectively.

**Conclusion:**

Considering the lower levels of CA-125 in patients with AA compared with NAA cases and also weak screening performance characteristics, it seems that it could not be considered as an accurate screening tool in this regard.

## Introduction:

Acute appendicitis (AA) is one of the common causes of abdominal emergency surgeries. The possibility of one facing this condition over their lifetime varies between 6.7% to 8.6% (1, 2). The accurate and timely diagnosis of acute appendicitis plays an essential role in preventing life-threatening complications such as perforation associated with other morbidities or mortality (3). 

The diagnosis of appendicitis is made based on clinical examination followed by laboratory and radiographic studies. These patients may undergo unnecessary hospital admissions and operations as the result of false-positive diagnosis, while the false-negative findings can lead to extreme consequences (4). 

Studies have shown that 40-83% of cases are detected based on the classic clinical symptoms (5-7). Currently, laboratory parameters such as white blood cell count, neutrophil percentage, and C-reactive protein concentration are used in various combinations to improve sensitivity and specificity of assessments for determining risk of appendicitis (8, 9). 

Carcinogen antigen 125 (CA-125) has shown potentials to be considered as a diagnostic test in this regard (10). CA-125 is a glycoprotein antigen, which is well known as a marker for epithelial ovarian cancer. Increased levels of CA-125 have been identified in benign and malignant conditions, including uterine leiomyoma, endometriosis, pelvic inflammatory disease, cirrhosis, and pleural or peritoneal effusion. Different types of coelomic epithelial cells such as peritoneal mesothelial cells have a role in producing CA-125, which may increase in conditions of peritoneal inflammation (11).

Some studies have applied CA-125 as a diagnostic tool in AA, which found the serum level of CA-125 to be higher in patients suffering from appendicitis (10, 12). However, some researchers opposed this notion with results indicating absence of a predictive role for this tumor marker (13, 14). According to the divergent findings, the association between CA-125 and AA is not well understood. The aim of this study is to determine the value of CA-125 in predicting AA.

## Methods:

***Study design and setting***

This prospective diagnostic accuracy study was conducted on patients with acute abdominal pain, suspected to AA, referred to emergency departments of Imam Reza and Sina University Hospitals, Tabriz, Iran, from 1^st^ of January 2016 to the end of December 2017. The research protocol was confirmed by the Ethics committee of Tabriz University of Medical Sciences (TBZMED.REC.94/3-7/23) and all the participants gave informed written consent prior to the study.

***Participants***

Using non-probability consecutive sampling method, the participants were chosen from patients between the ages of 15 to 70 years with acute abdominal pain suspected to acute appendicitis who were candidates for surgical appendectomy. Patients with history of smoking, diabetes, hypertension or any medical conditions that associated with increased level of serum CA-125, such as cirrhosis, congestive heart failure, inflammatory bowel disease, malignancy, endometriosis, pregnancy and recent abdominal surgery were excluded.

***Procedure***

The serum level of CA-125 was checked for all patients (using 5 cc blood sampling from left/right brachial vein) in the emergency department before the operation. An Electrochemiluminescence (ECL) assessment was done using E411 Cobas machine and kits produced by Germany Roche Company (based on Company reference) for CA-125 measurement. In addition, routine laboratory testing including white blood cell (WBC) level and polymorph nuclear (PMN) cell percentage were also done for each patient. Ultrasonography was also performed on each patient as part of the diagnostic approach. 

The gold standard for diagnosis of AA was positive histological findings described in pathology reports. Patients with an Alvarado score of 7 or above were considered for surgery.

***Data gathering***

A checklist consisting of patients’ demographic, clinical, and histopathological variables (after the surgery) was filled out for all patients by a senior emergency medicine resident under super vision of an emergency medicine specialist and supervisor of the project. 

The pathology reports were classified in three categories including negative for appendicitis, simple appendicitis or severe ones (phlegmonous, abscess, perforated or gangrenous appendix).

***Statistical analysis***

Sample size was calculated as 93 patients, considering 60% sensitivity, 100% specificity and a confidence interval of 95%, and 80% power. Statistical analysis was performed using SPSS software version 19.0 (IBM Corp., Armonk, N.Y., USA). Categorical variables were reported as percentages and continuous variables as mean ± standard deviation (SD). Continuous variables were compared using independent t-test and categorical variables were compared via chi-square test. Sensitivity, specificity, positive predictive value (PPV), negative predictive value (NPV), positive likelihood ratio (PLR), and negative likelihood ratio (NLR) as well as area under the receiver operating characteristics (ROC) curve of CA-125 in predicting AA were calculated using Medcalc software (version 18.2) and reported with 95% confidence interval (CI). P-values less than 0.05 were considered as significant. 

## Results:

95 patients with the mean age of 31.65 ± 12.9 (15-75) years were studied (54.3% male). Based on the histologic findings, 72 (75.8%) cases were categorized as AA (23 cases as severe) and the remaining 23 (24.2%) cases were normal (NAA). Table 1 compares the baseline characteristics of AA patients with others. The two groups were similar regarding mean age (p = 0.59), mean duration of symptoms (p = 0.08), mean white blood cell (WBC) count (p = 0.37), and mean PMN percentage (p = 0.55). 

**CA-125 level**

Mean CA-125 level was 16.5 ± 20.0 U/mL in the AA group and 30.5 ± 6.1 U/mL in the NAA group (p = 0.001). It was significantly higher in female patients (25.4 ± 31.8 vs 12.9 ± 10.7 U/mL; p = 0.03). Adjustment of analysis based on gender revealed a significant correlation between CA-125 level and diagnosis of AA only in females (34.23 ± 39 in NAA versus 20.7 ± 26.7 U/mL in AA, p = 0.012). Adjustment of analysis based on severity of AA revealed an insignificant correlation between severity of AA and CA-125 level (p = 0.058).

**Screening characteristics of CA-125**

**Figure 1:**
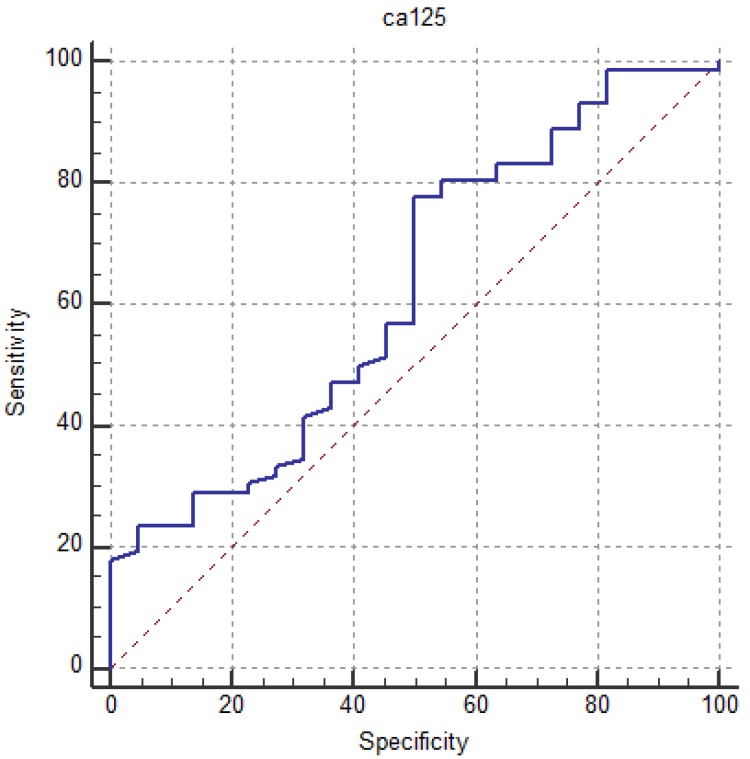
The area under the receiver operating characteristics (ROC) curve of CA-125 in predicting acute appendicitis

Figure 1 shows the area under the ROC curve of CA-125 in identifying cases with AA. The area under the ROC curve of CA-125 was 0.62 (95%CI: 0.51 to 0.72). Based on the ROC curve analysis, the best cut off point of CA-125 in predicting AA was estimated to be 16.4 U/mL. 

Sensitivity, specificity, NPV, PPV, NLR, and PLR of CA-125 in 16.4 U/mL cut off were 77.8% (95%CI: 66.4 - 86.7), 50.0% (95%CI: 28.2 - 71.8), 83.6% (95%CI: 76.7 - 88.7), and 40.7% (95%CI: 27.4 - 55.6), 0.44 (95%CI 0.2 - 0.8), and 1.56 (95%CI: 1.0 - 2.4), respectively.

## Discussion

In this study, we assessed the possible diagnostic value of CA-125 in detecting AA. We hypothesized that patients with AA have higher CA-125 levels compared with NAA patients. Surprisingly, NAA patients had higher CA-125 levels compared with AA patients. In the cut off value of 16.4 U/mL, CA-125 had a sensitivity of 77.8% and specificity of 50% in differentiating NAA patients from AA cases.

**Table 1:** Comparison of the baseline characteristics between patients with acute appendicitis and those with normal appendix on histologic findings

**Variable**	**Acute appendicitis**		**P value**
	**Yes (n=72)**	**No (n=23)**	
**Gender**			
Male	38 (52.7)	5 (21.7)	0.03
Female	34 (42.3)	18 (78.3)	
**Age (year)**			
Mean ± SD	37.25 ± 44.8	29.73 ± 14.9	0.59
**Duration of symptoms (hour)**			
Mean ± SD	12.53 ± 16.9	11.32 ± 18.2	0.08
**PMN (%)**			
Mean ± SD	75.1 ± 11.5	76.2 ± 12.2	0.55
**WBC count (/ mm** ^3^ **)**			
Mean ± SD	12022.3 ± 3871.6	10209.5 ± 4387.4	0.37

Data are reported as mean ± standard deviation (SD) or frequency (%). PMN: polymorph nuclear; WBC: white blood cell.

Literature supported that some tumor markers such as CA-125 may have additional value in not only the diagnosis of AA, but also the differentiation between severe complicated cases and simple cases (11). CA-125 is commonly used as a marker in gynecological cancers. Considering the possible role of peritoneal cells in secreting CA-125 during the phase of inflammation, this biomarker may increase over the course of peritonitis resulting from AA. Basaran et al. proposed that secretion of CA-125 starts six hours prior to the onset of inflammation (15). Zeimet et al. compared the release of CA-125 from peritoneal cells and malignant ovarian cells. They reported higher CA-125 synthesis in peritoneal cells due to inflammation (16). It is also proposed that CA-125 is secreted by apical surface of mesothelial monolayers as a response to inflammatory cytokines such as interleukin-1 beta and tumor necrotizing factor-alpha, and E coli lipopolysaccharide (17).

Berger et al. reported a significantly higher CA-125 level in males with severe appendicitis compared to simple cases, whereas, CA-125 in males with AA didn’t differ significantly from NAA patients. However, Sevinc et al. found a significant positive correlation between CA-125 levels and AA diagnosis, which also decreased after the surgical appendectomy (11).

 In contrast, in the present study, the serum levels of CA-125 in patients with severe AA did not significantly differ from simple types of disease, even after the categorization of the study population based on gender.

Cetinkaya et al. also examined the correlation between CA-125 and severe AA. They found high serum levels of CA-125 in severe AA cases with the cut off value of 35 U/mL. The calculated sensitivity, specificity, and PPVs were 60%, 100%, and 100%, respectively (18). 

In our study, the female group had significantly higher levels of CA-125. After analyzing CA-125 levels in gender specific subgroups, we found CA-125 levels to be higher in NAA cases compared to AA cases in the female subgroup. However, there was not a significant correlation between CA-125 levels and AA diagnosis in the male group. Secretion of CA-125 from ovarian cells in females may have affected the final results of the study. Some of the previous researchers also evaluated CA-125 levels only in male cases.

***Limitations***

The study population was limited to 95 patients and in studies with larger sample groups the results may differ from the present report. In addition, we did not evaluate CA-125 levels after the surgery to compare cases with AA and NAA. Hence, more studies with larger study populations and with higher cut off values are required to assess the possible role of CA-125 in AA diagnosis.

## Conclusion:

The results of the present study showed a low sensitivity and specificity for serum levels of CA-125 in differentiating patients with AA from those with NAA. Additionally, it was not an effective marker for diagnosis of the complicated form of AA. However, more research on a greater sample of patients is required to evaluate its predictive value.
